# Supplementary material for: Transient targeting of hypothalamic orexin neurons alleviates seizures in a mouse model of epilepsy
Source: Nat Commun. 2024 Feb 10;15:1249. doi: 10.1038/s41467-024-45515-5 (PMC10858876; doi:10.1038/s41467-024-45515-5)
Supplement: Supplementary file 1 — Supplementary Information [file 41467_2024_45515_MOESM1_ESM.pdf]

Supplementary Information

Transient targeting of hypothalamic orexin neurons alleviates seizures in a mouse model of epilepsy

Han-Tao Li[1,2], Paulius Viskaitis[1], Eva Bracey[1], Daria Peleg-Raibstein[1], Denis Burdakov[1*]

1. *Department of Health Sciences and Technology, Swiss Federal Institute of Technology | ETH Zurich, 8603 Schwerzenbach, Switzerland*
2. *Section of Epilepsy, Department of Neurology; Chang Gung Memorial Hospital at Linkou Medical Center and Chang Gung University College of Medicine, 333 Taoyuan, Taiwan*

Supplementary contents:

1. Supplementary Fig. 1: Effect of lateral hypothalamic DBS on sucrose preference test, forced swim test, appetite, mood/reward-related behaviours, and sleep pattern.
2. Supplementary Table 1: statistical table of all statistical results in figures and supplementary figure.

Supplementary Figure 1

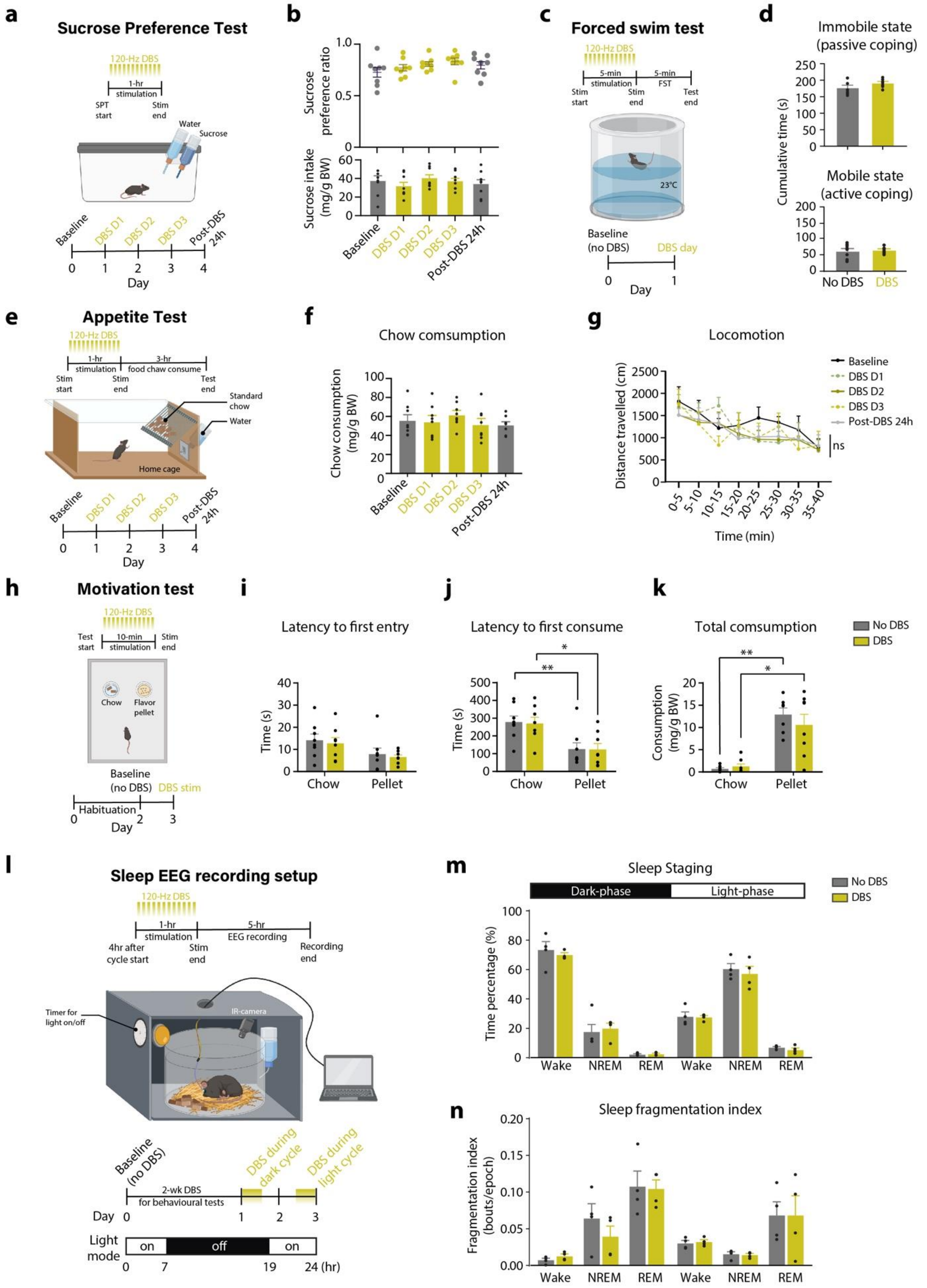

Supplementary Figure 1: Effect of lateral hypothalamic DBS on sucrose preference test, forced swim test, appetite, mood/reward-related behaviours, and sleep pattern.

A. Experimental protocol and paradigm for sucrose preference test before, during (day 1-3), and 24-hr after LH DBS.
B. Top, average sucrose preference ratio between groups (One-way RM ANOVA, $F_{(2.39,16.73)}$ = 1.168, P = 0.34). Bottom, total sucrose intake during the test (One-way RM ANOVA, $F_{(2.756,19.29)}$ = 0.5215, P = 0.66).
C. Experimental protocol and paradigm for forced swim test before, and after LH DBS.
D. Top, cumulative time of the immobility state (two-tailed t test, P = 0.12). Bottom, cumulative time of the mobility state (two-tailed t test, P = 0.72).
E. Experimental protocol and paradigm for appetite test before, during (day 1-3), and 24-hr after LH DBS.
F. Average chow between groups (One-way RM ANOVA, $F_{(2.105,14.74)}$ = 0.7439, P = 0.49).
G. Total distance travelled (5-min time bin) during the first 40 mins of appetite test (Two-way RM ANOVA, $F_{(4,280)}$ = 0.995, P = 0.41).
H. Experimental protocol and paradigm for motivation test before and during LH DBS. Inside the experimental chamber there are two food choice provided: standard food chow and chocolate flavor pellet.
I. Latency to first entry into different food zones (One-way RM ANOVA, $F_{(2.24,15.68)}$ = 2.546, P = 0.10).
J. Latency to first consume different food (One-way RM ANOVA, $F_{(1.82,12.75)}$ = 8.759, P = 0.005; Tukey's post-tests: chow-no DBS vs. pellet-no DBS, P = 0.009; chow-DBS vs. pellet-DBS, P = 0.03).
K. Total consumption of different food (One-way RM ANOVA, $F_{(1.78,12.46)}$ = 16.6, P = 0.0004; Tukey's post-tests: chow-no DBS vs. pellet-no DBS, P = 0.001; chow-no DBS vs. chow-DBS, P = 0.73; pellet-no DBS vs. pellet-DBS, P=0.96; chow-DBS vs. pellet-DBS, P = 0.04).
L. Experimental protocol of sleep recording chamber and experimental paradigm.
M. Average time percentage of different sleep stage (Wake, NREM, and REM) before and after DBS during dark- and light-phase (unpaired t test).
N. Sleep fragmentation index before and after DBS during dark- and light-phase (two-tailed t test, P values are all not significant).

Data are mean ± SEM of n = 8 mice for behavioral paradigms (panels a-k) and n = 4 mice for sleep EEG experiments. *P<0.05, **P<0.01. Cartoons in A,C,E,I are created with BioRender.

Supplementary Table 1: statistical table of all statistical results in figures and supplementary figure.

**Figure in which data are presented**	**Experimental variable**	**Data comparisons (experimental vs control)**	**n**	**Statistical test**	**P value**
Fig. 1C	Seizure EEG power	Seizure power across 5, 10 and 20Hz stimulation	6	One-way RM ANOVA	$F_{(2,10)}$=7.114, P=0.012; Tukey's post-tests: 5Hz vs. 20Hz: P=0.0096; : 5Hz vs. 10Hz: P=0.30; : 10Hz vs. 20Hz: P=0.12
Fig. 1D	Seizure probability	Pre-Vehicle vs. Pre-SB	6	24 stimulations	75 vs 83 %
		Post-Vehicle vs. Post-SB	6	24 stimulations	79 vs 54 %
	Seizure duration	Pre-Vehicle vs. Pre-SB	6	2-tailed unpaired t test	P=0.37, t=0.9331, df=10
		Post-Vehicle vs. Post-SB	6	2-tailed unpaired t test	P=0.61 t=0.5260, df=10
Fig. 1E	Seizure EEG power	Pre-Vehicle vs. Pre-SB	6	2-tailed unpaired t test	P=0.39, t=0.8805, df=10
		Post-Vehicle vs. Post-SB	6	2-tailed unpaired t test	P=0.03, t=2.462, df=10
Fig. 1F	Distance travelled	Pre-Vehicle vs. Pre-SB	6	2-tailed unpaired t test	P=0.54, t=0.6261, df=10
		Post-Vehicle vs. Post-SB	6	2-tailed unpaired t test	P=0.01, t=2.913, df=10
Fig. 2D	HON activity	Pre- vs. During-Sz	30	2-tailed paired t test	P= 0.001, t=3.345, df=58
Fig. 2E	Seizure EEG power	Actual vs. Predicated EEG power	30	Regression model	R square = 0.53
Fig. 2F	Relative contribution (%)	Pre- vs. During- vs. Post-Sz HON activity	30	2-tailed paired t test	p = 0.0001, t=4.070, df=58
Fig. 3D	Seizure EEG power	Control (Ct) 1 vs. Ct 2 vs. Sz ArchT vs. Pre-Sz ArchT	5	One-way ANOVA	$F_{(3,16)}$=3.842, P=0.03; Dunnett's post-tests: Ct 1 vs. Pre-Sz ArchT: P=0.02; Ct 1 vs. Ct 2: P>0.99; Ct1 vs ArchT at Seizure: P=0.79
	Seizure probability	Ct 1 vs. Ct 2 vs. Sz ArchT vs. Pre-Sz ArchT	5	18 stimulations	98 vs 95 vs 66 vs 33%
Fig. 3E	EEG power (seizure 1)	Ct 2 stim vs. Pre-Sz ArchT	5 vs 5	2-tailed unpaired t test	P=0.04, t=2.346, df=8
	EEG power (seizure 2)	Ct 2 stim vs. Pre-Sz ArchT	5 vs 5	2-tailed unpaired t test	P=0.96, t=0.049, df=8
	EEG power (seizure 3)	Ct 2 stim vs. Pre-Sz ArchT	5 vs 5	2-tailed unpaired t test	P=0.72, t=0.3736, df=8
	EEG power (seizure 4)	Ct 2 stim vs. Pre-Sz ArchT	5 vs 5	2-tailed unpaired t test	P=0.87, t=0.1729, df=8
Fig. 4C	Seizure EEG power	Baseline vs. No DBS vs. 120Hz sinusoid vs.	6	One-way ANOVA	$F_{(3,20)}$=5.382, P=0.007; Dunnett's post-tests: No DBS vs Baseline: P=0.08; No DBS vs 120Hz

		2Hz sinusoid DBS			sinusoid sinusoid: P=0.004; No DBS vs 2Hz sinusoid: P=0.75
Fig. 4D	Seizure probability	No DBS vs. 120Hz sinusoid vs. 2Hz sinusoid DBS	6	15 stimulations	83 vs 29 vs 75%
Fig. 5C	Seizure EEG power	No DBS vs. Pre-Sz DBS vs. DBS with Sz indurction vs. DBS after Sz induction	6	One-way ANOVA	$F_{(3,20)}$ = 6.936, P=0.002; Tukey's post-tests: No DBS vs.Pre-Sz DBS: P=0.01; No DBS vs.DBS at Sz induction: P=0.14; No DBS vs DBS after Sz induction: P=0.97; Pre-Sz DBS vs DBS at Sz induction: P=0.61; Pre-Sz DBS vs DBS after Sz induction: P=0.004; DBS at Sz induction vs DBS after Sz induction: P=0.06
Fig. 5D	EEG power (seizure 1)	Ct stim vs. Pre-Sz DBS	3 vs 3	2-tailed unpaired t test	P=0.01, t=4.531, df=4
	EEG power (seizure 2)	Ct stim vs. Pre-Sz DBS	3 vs 3	2-tailed unpaired t test	P=0.02, t=3.879, df=4
	EEG power (seizure 3)	Ct stim vs. Pre-Sz DBS	3 vs 3	2-tailed unpaired t test	P=0.003, t=6.67, df=4
	EEG power (seizure 4)	Ct stim vs. Pre-Sz DBS	3 vs 3	2-tailed unpaired t test	P=0.91, t=0.1205, df=4
Supp. Fig.1B	Sucrose preference test (sucrose preference ratio)	Baseline vs. DBS day1 vs. DBS day2 vs. DBS day3 vs. Post-DBS 24h	8	One-way RM ANOVA	$F_{(2.390,16.73)}$=1.168, P=0.34; Dunnett's post-tests: baseline vs. DBS D1: P=0.86; baseline vs. DBS D2: P=0.54; baseline vs. DBS D3: P=0.41; baseline vs. DBS D1: P=0.47
Supp. Fig.1B	Sucrose preference test (sucrose consumption)	Baseline vs. DBS day1 vs. DBS day2 vs. DBS day3 vs. Post-DBS 24h	8	One-way RM ANOVA	$F_{(2.756,19.29)}$=0.5215, P=0.66; Dunnett's post-tests: baseline vs. DBS D1: P=0.84; baseline vs. DBS D2: P=0.98; baseline vs. DBS D3: P>0.99; baseline vs. DBS D1: P=0.93
Supp. Fig.1D	Forced swim test (immobile)	No DBS vs. DBS	8	2-tailed unpaired t test	P=0.12, t=1.643, df=14
Supp. Fig.1D	Forced swim test (mobile)	No DBS vs. DBS	8	2-tailed unpaired t test	P=0.72, t=0.362, df=14
Supp. Fig.1F	Appetite test (chaw consumption)	Baseline vs. DBS day1 vs. DBS day2 vs. DBS day3 vs. Post-DBS 24h	8	One-way RM ANOVA	$F_{(2.105,14.74)}$=0.7439, P=0.49; Dunnett's post-tests: baseline vs. DBS D1: P=0.99; baseline vs. DBS D2: P=0.82;

					baseline vs. DBS D3: P=0.98; baseline vs. DBS D1: P=0.84
Supp. Fig.1G	Locomotion	Baseline vs. DBS day1 vs. DBS day2 vs. DBS day3 vs. Post-DBS 24h	8	Two-way ANOVA	$F_{(4,280)}$ = 0.995, P = 0.41
Supp. Fig.1I	Motivation test (Latency to first entry)	Chow vs. Pellet; No DBS vs. DBS	8	One-way RM ANOVA	$F_{(2.24,15.68)}$ = 2.546, P = 0.10; Tukey's post-tests: chow-no DBS vs. pellet-no DBS, P = 0.45; chow-no DBS vs. chow-DBS, P = 0.98; pellet-no DBS vs. pellet-DBS, P=0.97; chow-DBS vs. pellet-DBS, P = 0.16
Supp. Fig.1J	Motivation test (Latency to first eat)	Chow vs. Pellet; No DBS vs. DBS	8	One-way RM ANOVA	$F_{(1.82,12.75)}$ = 8.759, P = 0.005; Tukey's post-tests: chow-no DBS vs. pellet-no DBS, P = 0.009; chow-no DBS vs. chow-DBS, P = 0.99; pellet-no DBS vs. pellet-DBS, P>0.99; chow-DBS vs. pellet-DBS, P = 0.03
Supp. Fig.1K	Motivation test (Total consumption)	Chow vs. Pellet; No DBS vs. DBS	8	One-way RM ANOVA	$F_{(1.78,12.46)}$ = 16.6, P = 0.0004; Tukey's post-tests: chow-no DBS vs. pellet-no DBS, P = 0.001; chow-no DBS vs. chow-DBS, P = 0.73; pellet-no DBS vs. pellet-DBS, P=0.96; chow-DBS vs. pellet-DBS, P = 0.04
Supp. Fig.1M	Sleep staging	No DBS vs. DBS	4	2-tailed unpaired t test	Dark phase: wake, no DBS vs DBS, P = 0.58, t=0.5887, df=6; NREM, no DBS vs DBS, P = 0.75, t=0.3277, df=6; REM, no DBS vs DBS, P = 0.78, t=0.2856, df=6.<br>Light phase: wake, no DBS vs DBS, P = 0.90, t=0.1307, df=6; NREM, no DBS vs DBS, P = 0.62, t=0.5285, df=6; REM, no DBS vs DBS, P = 0.30, t=1.142, df=6.
Supp. Fig.1N	Sleep fragmentation index	No DBS vs. DBS	4	2-tailed unpaired t test	Dark phase: wake, no DBS vs DBS, P = 0.18, t=1.530, df=6; NREM, no DBS vs DBS, P = 0.35, t=1.021, df=6; REM, no DBS vs DBS, P = 0.90, t=0.1361, df=6.

					Light phase: wake, no DBS vs DBS, P = 0.68, t=0.4382, df=6; NREM, no DBS vs DBS, P = 0.73, t=0.3562, df=6; REM, no DBS vs DBS, P > 0.99, t=0.000, df=6
