# Supplementary material for: Transient targeting of hypothalamic orexin neurons alleviates seizures in a mouse model of epilepsy
Source: Nat Commun. 2024 Feb 10;15:1249. doi: 10.1038/s41467-024-45515-5 (PMC10858876; doi:10.1038/s41467-024-45515-5)
Supplement: Supplementary file 3 — Inventory of Supplementary Information [file 41467_2024_45515_MOESM3_ESM.docx]

Inventory of Supporting Information:

1. Supplementary Fig. 1: Effect of lateral hypothalamic DBS on sucrose preference test, forced swim test, appetite, mood/reward-related behaviours, and sleep pattern.
2. Supplementary Table 1: statistical table of all statistical results in figures and supplementary figure.
