# Supplementary material for: Transient targeting of hypothalamic orexin neurons alleviates seizures in a mouse model of epilepsy
Source: Nat Commun. 2024 Feb 10;15:1249. doi: 10.1038/s41467-024-45515-5 (PMC10858876; doi:10.1038/s41467-024-45515-5)
Supplement: Supplementary file 4 — Reporting Summary [file 41467_2024_45515_MOESM4_ESM.pdf]

nature portfolio

Corresponding author(s): Denis Burdakov

Last updated by author(s): Jan 5, 2024

Reporting Summary

Nature Portfolio wishes to improve the reproducibility of the work that we publish. This form provides structure for consistency and transparency in reporting. For further information on Nature Portfolio policies, see our Editorial Policies and the Editorial Policy Checklist.

Statistics

For all statistical analyses, confirm that the following items are present in the figure legend, table legend, main text, or Methods section.

n/a	Confirmed	
☐	☒	The exact sample size (*n*) for each experimental group/condition, given as a discrete number and unit of measurement
☐	☒	A statement on whether measurements were taken from distinct samples or whether the same sample was measured repeatedly
☐	☒	The statistical test(s) used AND whether they are one- or two-sided<br>*Only common tests should be described solely by name; describe more complex techniques in the Methods section.*
☐	☒	A description of all covariates tested
☐	☒	A description of any assumptions or corrections, such as tests of normality and adjustment for multiple comparisons
☐	☒	A full description of the statistical parameters including central tendency (e.g. means) or other basic estimates (e.g. regression coefficient) AND variation (e.g. standard deviation) or associated estimates of uncertainty (e.g. confidence intervals)
☐	☒	For null hypothesis testing, the test statistic (e.g. *F*, *t*, *r*) with confidence intervals, effect sizes, degrees of freedom and *P* value noted<br>*Give P values as exact values whenever suitable.*
☒	☐	For Bayesian analysis, information on the choice of priors and Markov chain Monte Carlo settings
☒	☐	For hierarchical and complex designs, identification of the appropriate level for tests and full reporting of outcomes
☒	☐	Estimates of effect sizes (e.g. Cohen's *d*, Pearson's *r*), indicating how they were calculated

Our web collection on statistics for biologists contains articles on many of the points above.

Software and code

Policy information about availability of computer code

Data collection	EEG/EMG and photometry data: Spike2 software via a CED Micro1401-3 data acquisition unit (Cambridge Electronic Design, Cambridge, UK). Video tracking: Noldus Ethovision XT 16.
Data analysis	Raw data was processed in MATLAB 2019b (Mathworks, USA). Statistical analysis was done using Prism 9 (GraphPad Software Inc, California, USA). Figshare link with codes: https://doi.org/10.6084/m9.figshare.24167898

For manuscripts utilizing custom algorithms or software that are central to the research but not yet described in published literature, software must be made available to editors and reviewers. We strongly encourage code deposition in a community repository (e.g. GitHub). See the Nature Portfolio guidelines for submitting code & software for further information.

Data

Policy information about availability of data

All manuscripts must include a data availability statement. This statement should provide the following information, where applicable:

- Accession codes, unique identifiers, or web links for publicly available datasets
- A description of any restrictions on data availability
- For clinical datasets or third party data, please ensure that the statement adheres to our policy

Here is the data availability statement included in the manuscript: "DATA AVAILABILITY
The data generated in this study have been deposited in the Figshare repository (https://doi.org/10.6084/m9.figshare.24167898)78. Source data are provided with

April 2023

this paper.

Research involving human participants, their data, or biological material

Policy information about studies with human participants or human data. See also policy information about sex, gender (identity/presentation), and sexual orientation and race, ethnicity and racism.

Reporting on sex and gender	N/A
Reporting on race, ethnicity, or other socially relevant groupings	N/A
Population characteristics	N/A
Recruitment	N/A
Ethics oversight	N/A

Note that full information on the approval of the study protocol must also be provided in the manuscript.

Field-specific reporting

Please select the one below that is the best fit for your research. If you are not sure, read the appropriate sections before making your selection.

☒ Life sciences ☐ Behavioural & social sciences ☐ Ecological, evolutionary & environmental sciences

For a reference copy of the document with all sections, see nature.com/documents/nr-reporting-summary-flat.pdf

Life sciences study design

All studies must disclose on these points even when the disclosure is negative.

Sample size	Sample sizes were kept to a minimum to be compliant with local mouse usage policies, but were consistent with those used in recent literature using similar techniques and experimental models (e.g. these papers: https://pubmed.ncbi.nlm.nih.gov/28334611/, https://pubmed.ncbi.nlm.nih.gov/25703096/)
Data exclusions	No data were excluded from the analyses.
Replication	All attempts at replication were successful. The number of replication is defined as n number and compiled in Supplementary Table 1.
Randomization	Each animal was randomly assigned to an experimental group in all experiments.
Blinding	Blinding was not relevant in most figures since data collection and analyses were automated; in the small number of cases where manual video-scoring was performed, investigators were blinded to treatment groups.

Reporting for specific materials, systems and methods

We require information from authors about some types of materials, experimental systems and methods used in many studies. Here, indicate whether each material, system or method listed is relevant to your study. If you are not sure if a list item applies to your research, read the appropriate section before selecting a response.

Materials & experimental systems

n/a	Involved in the study	
☒	☐	Antibodies
☒	☐	Eukaryotic cell lines
☒	☐	Palaeontology and archaeology
☐	☒	Animals and other organisms
☒	☐	Clinical data
☒	☐	Dual use research of concern
☒	☐	Plants

Methods

n/a	Involved in the study	
☒	☐	ChIP-seq
☒	☐	Flow cytometry
☒	☐	MRI-based neuroimaging

Animals and other research organisms

Policy information about studies involving animals; ARRIVE guidelines recommended for reporting animal research, and Sex and Gender in Research

Laboratory animals	Subjects were adult male C57BL/6 mice, over the age of eight weeks.
Wild animals	The study did not involve wild animals.
Reporting on sex	Sex and gender were not specifically considered in the study design; this study only involved male mice, this is stated and acknowledged.
Field-collected samples	The study did not involve samples collected from the field.
Ethics oversight	All experiments were performed following the Swiss Federal Food Safety and Veterinary Office Welfare Ordinance (TSchV 455.1, approved by the Zurich Cantonal Veterinary Office).

Note that full information on the approval of the study protocol must also be provided in the manuscript.
